# Bimolecular Rate Constants for FAD-Dependent Glucose Dehydrogenase from *Aspergillus terreus* and Organic Electron Acceptors

**DOI:** 10.3390/ijms18030604

**Published:** 2017-03-10

**Authors:** Nozomu Tsuruoka, Takuya Sadakane, Rika Hayashi, Seiya Tsujimura

**Affiliations:** Division of Materials Science, Faculty of Pure and Applied Sciences, University of Tsukuba, 1-1-1 Tennodai, Tsukuba, Ibaraki 305-8573, Japan; nozomu.tsuruoka1@gmail.com (N.T.); takumegu0804@gmail.com (T.S.); hayashi.rika.gf@un.tsukuba.ac.jp (R.H.)

**Keywords:** flavin adenine dinucleotide, glucose dehydrogenase, phenothiazine, quinone, redox mediator

## Abstract

The flavin adenine dinucleotide-dependent glucose dehydrogenase (FAD-GDH) from *Aspergillus* species require suitable redox mediators to transfer electrons from the enzyme to the electrode surface for the application of bioelectrical devices. Although several mediators for FAD-GDH are already in use, they are still far from optimum in view of potential, kinetics, sustainability, and cost-effectiveness. Herein, we investigated the efficiency of various phenothiazines and quinones in the electrochemical oxidation of FAD-GDH from *Aspergillus terreus*. At pH 7.0, the logarithm of the bimolecular oxidation rate constants appeared to depend on the redox potentials of all the mediators tested. Notably, the rate constant of each molecule for FAD-GDH was approximately 2.5 orders of magnitude higher than that for glucose oxidase from *Aspergillus* sp. The results suggest that the electron transfer kinetics is mainly determined by the formal potential of the mediator, the driving force of electron transfer, and the electron transfer distance between the redox active site of the mediator and the FAD, affected by the steric or chemical interactions. Higher *k*_2_ values were found for ortho-quinones than for para-quinones in the reactions with FAD-GDH and glucose oxidase, which was likely due to less steric hindrance in the active site in the case of the ortho-quinones.

## 1. Introduction

Flavin adenine dinucleotide-dependent glucose dehydrogenases (FAD-GDHs) have recently garnered considerable attention as bioelectrocatalysts for glucose biosensors and biofuel cells because of their unique characteristics, including oxygen insensitivity, high biocatalytic activity, and substrate specificity [1,2,3]. In fact, FAD-GDHs isolated from several *Aspergillus* species have already been employed as catalysts in disposable blood glucose sensor strips, indicating that they may be promising alternatives to glucose oxidases (GOxs) [4]. Although some bacterial FAD-GDHs have the ability to transfer electrons directly from the enzyme to the surface of electrode [5,6], FAD-GDHs from *Aspergillus* species require redox mediators to shuttle electrons from the FAD site to the electrode [2,3]. For example, hexacyanoferrate ([Fe(CN)_6_]^3−^; ferricyanide) has been used as a redox mediator in commercially available FAD-GDH-based blood glucose sensor strips because of its high solubility in water, low cost, and high stability [2,4]. However, the reactivity between FAD-GDH and ferricyanide is quite low, where the bimolecular rate constant (*k*_2_ = *k*_cat_/*K*_M_) has been documented to be as low as 1 × 10^3^ M^−1^·s^−1^ in phosphate buffer (pH 7.0) at room temperature [2]. This low kinetic constant could be attributed to the low affinity between the active site of FAD-GDH and ferricyanide (i.e., an immeasurably high Michaelis–Menten constant). For the further development and performance improvement of bioelectrochemical devices, selection of a suitable mediator is critical. A mediator that has a low redox potential, but exhibits high electron transfer activity, is desired for high-power biofuel cells and for highly sensitive biosensors which is not affected by interfering substances such as uric acid or ascorbic acid [7,8,9].

Osmium (Os) complexes, a group of metal complex-type redox mediators used with GOxs, appear to react much more efficiently with FAD-GDH [3,10,11]. Osmium complex-type mediators are useful because, in addition to maintaining high kinetic constants through their efficient self-exchange electron transfer capabilities, they make it possible to control the potential at which the reaction proceeds solely by replacing the ligand [7,12,13,14,15,16]. However, Os complexes are expensive and non-sustainable, rendering them impractical for use in low-cost and disposable FAD-GDH-based electrodes. Thus, there is a need to select or identify other organic compounds or metal complexes based on earth abundant metals that can efficiently mediate redox reactions with FAD-GDH while also being economically viable for low-cost bioelectrical devices. Recently, naphthoquinone and the naphthoquinone-based redox polymer hydrogel were applied to an FAD-GDH-based electrode to improve its biofuel cell performance [17]. Naphthoquinone/phenanthrenequinone derivatives have also been investigated for their application in blood glucose sensors [18,19]. The choice of mediator is very important as it determines the potential at which the reaction proceeds, as well as the reaction rate (current density). However, it is not easy to obtain information on the optimum properties of potential mediators for FAD-GDHs. Even with structural and chemical information about the active center pocket obtained by crystal structure analysis of the enzyme, it would be difficult to predict the suitable mediator to use. Although there is an increasing number of reports on the discovery and recombinant production of new FAD-GDHs from various fungi [20,21,22,23,24,25,26], it is not realistic from the viewpoint of time and cost to clarify the mediator structure for all these newly discovered enzymes. Therefore, in this study, we propose a strategy for determining the optimum mediator for FAD-GDHs by screening a set of organic redox mediators via their bimolecular rate constants for the enzyme reaction. For this purpose, a set of quinones and phenothiazines (Table 1) were used, since their structure and redox potentials can be easily tuned by changing the substituents. The findings of this study may be useful as the starting point for the retrosynthetic analysis of new mediators.

## 2. Results and Discussion

**Figure 1:**
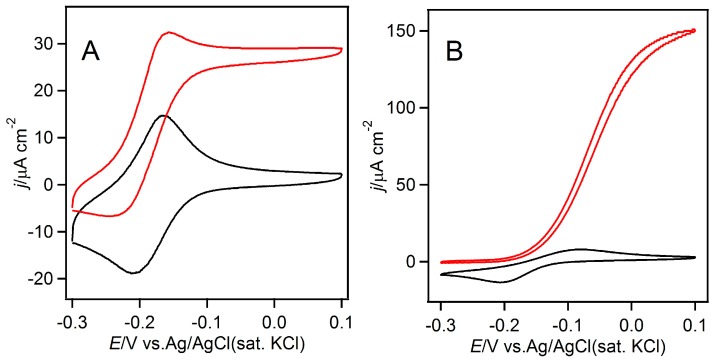
Cyclic voltammograms of (**A**) 0.1 mM toluidine blue and(**B**) 0.1 mM 1,4-naphthoquinone in the presence (red) or absence (black) of FAD-GDH (1.0 μM) in a phosphate buffer (pH 7.0) containing 0.1 M glucose. Scan rate of 10·mV·s^−1^.

In this study, an electrochemical method was applied to evaluate the bimolecular rate constants of the selected electron acceptors for FAD-GDH and GOx. Since the difference in the extinction coefficients of oxidized and reduced quinones is quite small, a color change measurement was not adequate for the kinetic analyses. Furthermore, the solubility of some quinones is so low that it is difficult to increase their concentration to observe the maximum turnover rate (*k*_cat_ value). We first confirmed the electrochemical behavior of the electron acceptors by cyclic voltammetry. Figure 1 shows the cyclic voltammograms (CVs) of 0.1 mM toluidine blue (TB) and 0.1 mM 1,4-naphthoquinone (14NQ), which were recorded in the presence or absence of FAD-GDH in phosphate buffer (pH 7) containing glucose. From these CVs, the formal potentials (i.e., mid-point potential of oxidation and reduction peak potentials) of TB and 14NQ were evaluated to be −0.19 and −0.15 V, respectively, vs. Ag/AgCl (sat. KCl). In this same way, the formal potentials of all the other chemicals were evaluated and are listed in Table 1. In the presence of FAD-GDH in the glucose-containing phosphate buffer, clear glucose oxidation currents were observed (Figure 1). Typical sigmoidal curves were observed for both TB and 14NQ, but the steady-state catalytic current density and the potential at which the catalytic current reached the steady state differed. The catalytic current density depended on the oxidation kinetics of the enzyme, as determined by the electron acceptors (details discussed in the following section).

**Figure 2:**
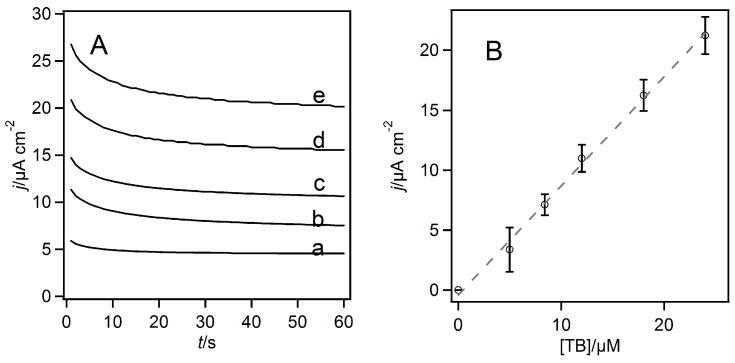
(**A**) Dependence of the current density on the constant potential electrolysis time for various toluidine blue (TB) concentrations; curves (a) 5.0 μM, (b) 8.4 μM, (c) 12 μM, (d) 16 μM, and (e) 24 μM TB; (**B**) Dependence of the steady-state catalytic current on the TB concentration. The error bars were evaluated by a Student’s *t*-distribution at a 90% confidence level. Dashed lines represent the regression line considering the weight of the error.

**Figure 3:**
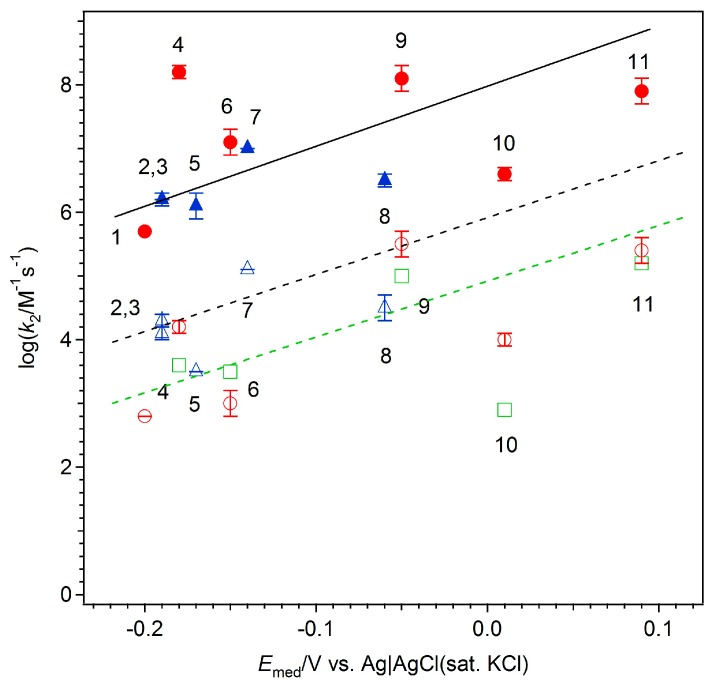
Dependence of the logarithmic bimolecular rate constants for FAD-GDH (closed symbols) and glucose oxidase (open symbols) on the formal potential of the mediators at pH 7.0: MeNQ (1), TB (2), AA (3), PQ (4), MB (5), 14NQ (6), TH (7), MG (8), 12NQ (9), 12NQS (10), and BQ (11). (See Table 1 for the abbreviation definitions.) The error bars were evaluated by a Student’s *t*-distribution at a 90% confidence level. Phenothiazines are depicted as blue triangles, and quinones are depicted as red circles. The glucose oxidase from the *Penicillium vitale*–quinone system is shown as green squares [27]. Black solid, black dashed, and green dashed lines represent the regression lines for FAD-GDH from *A. terreus* and GOx from *A.* sp. and GOx from *P. vitale* [27].

Based on the CVs of the phenothiazines and quinones examined in the presence of the enzyme and glucose, we were able to determine the applied potential for the constant potential amperometry (chronoamperometry) for measuring the steady-state catalytic glucose oxidation current. Figure 2A shows the TB-mediated FAD-GDH-catalyzed reaction current density (*j*) at 0.05 V vs. Ag/AgCl (sat. KCl) as a function of the electrolysis time for various TB concentrations. The steady-state catalytic current density increased linearly with increasing TB concentration, as evident in Figure 2B. By using these chronoamperometry readings of the dependence of the steady-state *j* on the mediator concentration, the bimolecular kinetic constants for FAD-GDH oxidation by the phenothiazines and quinones (*k*_2_ = *k*_cat_/*K*_M_) could be evaluated from the following equation [28]:

(1) $$j=2F\sqrt{\frac{D_{M}k_{\mathrm{cat}}[E]}{K_{M}}}[M]$$

where *F*, *D*_M_, [E], and [M] are the Faraday constant, diffusion coefficient of the mediator, concentration of the enzyme, and concentration of the mediator, respectively. Figure 3 shows the dependence of the logarithmic bimolecular rate constants (*k*_2_ = *k*_cat_/*K*_M_) on the formal potential of the phenothiazines (blue triangles) and quinones (red circles) for FAD-GDH (closed symbols) and for GOx (open symbols).

For the GOx from the *Penicillium vitale*–quinone system (green squares in Figure 3), the regression line for the logarithmic *k*_2_ values as a function of the formal potential of the mediators was analyzed on the basis of the Marcus theory (i.e., in terms of the self-exchange rate constants of the enzyme and mediators); log *k*_2_ (M^−1^·s^−1^) = 4.9 + 8.4 × *E*_med_ (modified from the original paper on reference electrodes) [27,29]. In the −0.2 to 0.1 V range, the logarithm of each *k*_2_ value for FAD-GDH and those for the GOx from the *Aspergillus* species also increased linearly as a function of the formal potential of the phenothiazines and quinones. The regression lines for FAD-GDH (solid line) and GOx (black dashed line), as shown in Figure 3, can be represented roughly by the following equations, based on the GOx (from *P. vitale*)-mediator system [27]:

log (*k*_2_ for FAD-GDH (M^−1^·s^−1^)) = 8.1 + 8.4 × *E*_med_ (2)

log (*k*_2_ for GOx from *Aspergillus* sp. (M^−1^·s^−1^)) = 5.5 + 8.4 × *E*_med_ (3)

The results suggest that electron transfer from the reduced FAD active site—found deep within the enzyme—is strongly governed by the outer-sphere electron transfer mechanism. Interestingly, the kinetic parameter did not depend on whether the mediator was a quinone or a phenothiazine, but instead depended mainly on the formal potential of the molecules. Overall, we observed that the bimolecular rate constant of each mediator was higher for FAD-GDH than for GOx by approximately 2.5 orders of magnitude. It is possible that this difference is related to the structural differences between these two enzymes. GOx has a homodimeric structure with a narrow active site pocket, resulting in a minimum distance of 13 Å between the protein surface and the FAD site [30,31]. In contrast, according to the crystal structure of FAD-GDH from *Aspergillus flavus* [32], this enzyme has a broader substrate-binding pocket than that of GOx, which allows easier access of redox molecules to the FAD site. The bimolecular rate constant of pyrroloquinoline quinone-dependent glucose dehydrogenase (PQQ-GDH) for a variety of quinones showed similar potential-dependent behavior, although PQQ-GDH exhibits potential-dependent activation-controlled as well as potential-independent diffusion-controlled regions [33]. The *k*_2_ value of PQQ-GDH in the potential-dependent region was 2 orders of magnitude higher than the maximum *k*_2_ value of FAD-GDH, which suggests that the difference could be due to the structure of the active site pocket.

The long-range electron transfer rate also depends on the electron transfer distance, which would be affected by the accessibility of the molecule to the FAD site, which is the closest transfer distance [30,31]. 9,10-Phenanthrenequinone (PQ) showed higher *k*_2_ values than expected from the regression line, which could be due to some attractive forces between the active site and the molecule, including π–π interactions (π stacking), π–cation interactions, or hydrophobic interactions, as well as the ortho-quinone structure, which is favorable for electron transfer [17]. On the contrary, the rate constants for the bimolecular reaction of 2-methyl-1,4-naphthoquinone (MeNQ), methylene green (MG), and 1,2-naphthoquinone-4-sulfonate (NQS) with FAD-GDH and GOx were lower than expected from the potential-dependent calibration line. This could be due to steric hindrance, since the functional groups of these molecules (a methyl group in MeNQ, a nitro group in MG, and a sulfo group in NQS) are close to the redox active site and could therefore potentially disturb the access to FAD. However, the rate constant for 1,4-benzoquinone (BQ), whose molecular structure is the smallest among the molecules examined, was lower than expected from the calibration line. This might be due to the inverted region of the Marcus theory or to the weak interactions (or a lack of favorable interactions) between BQ and the active site of the enzymes. Intriguingly, among all the molecules used in this study, only BQ lacked an aromatic ring.

The higher catalytic currents seen with FAD-GDH, in spite of the low formal potential, was likely due to the decreased steric hindrance in terms of access to the FAD active site compared with that for GOx. With regard to the formal potential, higher *k*_2_ values were found for ortho-quinones (including PQ and 1,2-naphthoquinone) than for para-quinones in the reactions with FAD-GDH and GOx. Thus, the mechanism for effective electron transfer could be explained by steric hindrance (i.e., the electron transfer distance between the FAD site and the redox site of the quinone molecules).

## 3. Materials and Methods

### 3.1. Materials

FAD-GDH from *Aspergillus terreus* was kindly donated by Ikeda Tohka Industries Co., Ltd. (Fukuyama, Japan) [2]. The GOx from *Aspergillus* species was purchased from Toyobo (product code GLO-201; Osaka, Japan). The FAD-GDH and GOx concentrations were determined spectrophotometrically using a molar extinction coefficient of free FAD of 11.3 × 10^3^ M^−1^·cm^−1^ at 465 nm and 13.1 × 10^3^ M^−1^·cm^−1^ at 450 nm, respectively [2]. The following phenothiazines were used in this study: thionine acetate (Wako Pure Chemical Industries, Osaka, Japan), azureA chloride (Sigma-Aldrich, St. Louis, MO, USA), basic blue17 (toluidine blue; Tokyo Chemical Industry Co., Tokyo, Japan), methylene blue hydrate (Tokyo Chemical Industry Co.), and basic green 5 (methylene green; Tokyo Chemical Industry Co.). The following quinones were used in this study and were purchased from Wako Pure Chemical Industries: 1,4-benzoquinone, 1,2-naphthoquinone, 1,2-naphthoquinone-4-sulfonate, 1,4-naphthoquinone, 2-methyl-1,4-naphthoquinone, and 9,10-phenanthrenequinone. All materials were used without further purification. Table 1 illustrates the structures of the phenothiazines and quinones in their oxidized forms. All had a redox potential in the range of −0.20–0.09 V at pH 7.0 relative to an Ag/AgCl (sat. KCl) reference electrode.

### 3.2. Homogeneous Redox Reactions of FAD-GDH with the Electron Acceptors

A gold (Au) electrode, 3 mm in diameter, was polished with an alumina slurry and rinsed with distilled water. Electrochemical measurements were performed using an electrochemical analyzer (BAS CV 50 W; BAS Inc., West Lafayette, IN, USA), with a platinum wire and an Ag/AgCl (sat. KCl) electrode as the counter and reference electrodes, respectively. Cyclic voltammetry of the redox molecules (0.1 mM) was carried out in phosphate buffer (0.1 M, pH 7) containing glucose (0.1 M), in the presence or absence of FAD-GDH (1.0 μM), at a scan rate of 10 mV·s^−1^. Chronoamperometry for the kinetic analysis was carried out at the potential where the steady-state current was observed, in phosphate buffer (0.1 M) containing FAD-GDH (1.0 μM) or GOx (5.0 μM), and glucose (0.1 M). For the kinetic analysis, the diffusion coefficient of each mediator (0.1 mM) was evaluated from the dependence of its plateau current value on the electrode rotation rate (1000, 2000, 3000, 4000, and 5000 rpm), using the Levich equation (Table 1). All measurements were carried out at room temperature (25 ± 1 °C). Prior to each experiment, the solutions were bubbled with Argon (Ar) for 10 min. Glucose stock solutions were stored overnight in order to achieve mutarotative equilibrium.

## 4. Conclusions

The bimolecular rate constants for FAD-GDH oxidation by phenothiazines and quinones at pH 7.0 varied between 10^6^ and 10^8^ M^−1^·s^−1^ depending on the formal potential of the redox mediator. The overall results suggest that the kinetics determined by the formal potential of the mediator, and the electron transfer distance between the redox active site of the molecules and the FAD site of the glucose dehydrogenase, greatly affect the electron transfer rate. More specifically, ortho-type redox active sites with π-electrons are favorable, whereas bulky functional groups, especially hydrophilic (charged) functional groups, close to the redox site are unfavorable.

There is a strong need for more cost-effective mediators for the bioelectrochemical application of FAD-GDH that can allow high biocatalytic activity at low catalyst-mediator overpotential. The choice of a mediator is very important, as it determines the potential at which the reaction proceeds as well as the reaction rate. Further kinetic analysis and spectroscopic analysis, in combination with structural information about the active site, will no doubt provide a clearer understanding of the factors governing the mediator reactions. In the meantime, our kinetic analysis strategy using a set of quinones and phenothiazines provides an important guideline for the identification, potential design, and selection of more efficient redox mediators for FAD-GDH. The approach presented here can also be used to fully exhibit the performances of other new or mutant FAD-GDHs with minimized kinetic and steric hindrances.

## Figures and Tables

**Table 1:** Redox mediators and the enzyme oxidation rates measured at pH 7 and 25 °C.

Compound	*E* (V vs. Ag/AgCl (sat. KCl))	Structure	*D*_M_ × 10^6^ (cm^2^·s^−1^)	log (*k*_2_ for FAD-GDH/M^−1^·s^−1^)	log (*k*_2_ for GOx/M^−1^·s^−1^)
2-Methyl-1,4-naphthoquinone (MeNQ)	−0.20	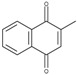	7.0	5.7 ± 0.0	2.8
Toluidine blue (TB)	−0.19	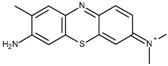	2.4	6.2 ± 0.1	4.3 ± 0.1
AzureA (AA)	−0.19	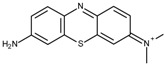	3.6	6.2 ± 0.0	4.1 ± 0.1
9,10-Phenanthrenequinone (PQ)	−0.18	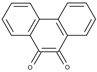	6.0	8.2 ± 0.1	4.2 ± 0.1 3.6 *
Methylene blue (MB)	−0.17	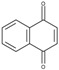	4.3	6.1 ± 0.2	3.5 ± 0.0
1,4-Naphthoquinone (14NQ)	−0.15	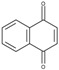	6.1	7.1 ± 0.2	3.0 ± 0.2 3.5 *
Thionine (TH)	−0.14	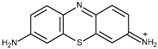	2.6	7.0 ± 0.0	5.1 ± 0.0
Methylene green (MG)	−0.06	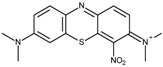	4.3	6.5 ± 0.1	4.5 ± 0.2
1,2-Naphthoquinone (12NQ)	−0.05	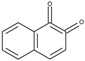	6.1	8.1 ± 0.2	5.5 ± 0.2 5.0 *
1,2-Naphthoquinone-4-sulfonate (NQS)	0.01	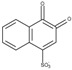	3.0	6.6 ± 0.1	4.0 ± 0.1 2.9 *
1,4-Benzoquinone (BQ)	0.09	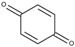	8.4	7.9 ± 0.2	5.4 ± 0.2 5.2 *

* From J. Kuly, N. Cenas, *Biochim. Biophys. Acta* **1983**, *744*, 57–63 [27]. GOx = glucose oxidase; FAD-GDH = flavin adenine dinucleotide-dependent glucose dehydrogenasese; *D*_M_ = diffusion coefficient of the mediator; *E* = formal potential of the mediator; *k*_2_ = oxidation rate constant of the mediator.
